# Humoral Response Following 3 Doses of mRNA COVID-19 Vaccines in Patients With Non-Dialysis-Dependent CKD: An Observational Study

**DOI:** 10.1177/20543581231224127

**Published:** 2024-01-29

**Authors:** Omosomi Enilama, Kevin Yau, Lee Er, Mohammad Atiquzzaman, Matthew J. Oliver, Marc G. Romney, Jerome A. Leis, Kento T. Abe, Freda Qi, Karen Colwill, Anne-Claude Gingras, Michelle A. Hladunewich, Adeera Levin

**Affiliations:** 1 Experimental Medicine, Department of Medicine, The University of British Columbia, Vancouver, Canada; 2 Nephrology Research Program, Providence Research, Vancouver, BC, Canada; 3 Division of Nephrology, Department of Medicine, Sunnybrook Health Sciences Centre, Toronto, ON, Canada; 4 Division of Nephrology, Department of Medicine, Unity Health Toronto, ON, Canada; 5 BC Renal, Vancouver, BC, Canada; 6 Ontario Renal Network, Toronto, ON, Canada; 7 Department of Pathology and Laboratory Medicine, St. Paul’s Hospital, Providence Health Care, Vancouver, BC, Canada; 8 Department of Pathology and Laboratory Medicine, Faculty of Medicine, The University of British Columbia, Vancouver, Canada; 9 Division of Infectious Diseases, Sunnybrook Health Sciences Centre, Toronto, ON, Canada; 10 Department of Molecular Genetics, University of Toronto, ON, Canada; 11 Lunenfeld-Tanenbaum Research Institute, Mount Sinai Hospital, Sinai Health System, Toronto, ON, Canada; 12 Division of Nephrology, The University of British Columbia, Vancouver, Canada; 13 St. Paul’s Hospital, Vancouver, BC, Canada

**Keywords:** COVID-19, serology, vaccine, chronic kidney disease, non-dialysis-dependent

## Abstract

**Background:**

Chronic kidney disease (CKD) is associated with a lower serologic response to vaccination compared to the general population. There is limited information regarding the serologic response to coronavirus disease 2019 (COVID-19) vaccination in the non-dialysis-dependent CKD (NDD-CKD) population, particularly after the third dose and whether this response varies by estimated glomerular filtration rate (eGFR).

**Methods:**

The NDD-CKD (G1-G5) patients who received 3 doses of mRNA COVID-19 vaccines were recruited from renal clinics within British Columbia and Ontario, Canada. Between August 27, 2021, and November 30, 2022, blood samples were collected serially for serological testing every 3 months within a 9-month follow-up period. The severe acute respiratory syndrome coronavirus 2 (SARS-CoV-2) anti-spike, anti-receptor binding domain (RBD), and anti-nucleocapsid protein (NP) levels were determined by enzyme-linked immunosorbent assay (ELISA).

**Results:**

Among 285 NDD-CKD patients, the median age was 67 (interquartile range [IQR], 52-77) years, 58% were men, 48% received BNT162b2 as their third dose, 22% were on immunosuppressive treatment, and COVID-19 infection by anti-NP seropositivity was observed in 37 of 285 (13%) patients. Following the third dose, anti-spike and anti-RBD levels peaked at 2 months, with geometric mean levels at 1131 and 1672 binding antibody units per milliliter (BAU/mL), respectively, and seropositivity rates above 93% and 85%, respectively, over the 9-month follow-up period. There was no association between eGFR or urine albumin-creatinine ratio (ACR) with mounting a robust antibody response or in antibody levels over time. The NDD-CKD patients on immunosuppressive treatment were less likely to mount a robust anti-spike response in univariable (odds ratio [OR] 0.43, 95% confidence interval [CI]: 0.20, 0.93) and multivariable (OR 0.52, 95% CI: 0.25, 1.10) analyses. An interaction between age, immunoglobulin G (IgG) antibody levels, and time was observed in both unadjusted (anti-spike: *P* = .005; anti-RBD: *P* = .03) and adjusted (anti-spike: *P* = .004; anti-RBD: *P* = .03) models, with older individuals having a more pronounced decline in antibody levels over time.

**Conclusion:**

Most NDD-CKD patients were seropositive for anti-spike and anti-RBD after 3 doses of mRNA COVID-19 vaccines and we did not observe any differences in the antibody response by eGFR.

## Background

The novel severe acute respiratory syndrome coronavirus 2 (SARS-CoV-2), which causes coronavirus disease 2019 (COVID-19), has been a major cause of morbidity and mortality worldwide since December 2019.^1^ Several studies have shown that the older adults and people with kidney disease are at an increased risk of COVID-19-related mortality, hospitalization, and adverse outcomes.^2-4^ Chronic kidney disease (CKD) is marked by chronic inflammation and immune dysfunction that usually results in lower rates of seroconversion, lower antibody levels, and a less sustained humoral response to vaccination compared with the general population.^5-7^ In addition, some studies have demonstrated a graded antibody response by estimated glomerular filtration rate (eGFR) in CKD patients after hepatitis B vaccination.^8,9^ The BNT162b2 Pfizer-BioNTech and mRNA-1273 Moderna COVID-19 vaccines were found to be highly efficacious against ancestral SARS-CoV-2 in randomized controlled trials but largely excluded patients with CKD or on immunosuppressive treatment.^10,11^

To date, a limited number of studies have investigated the COVID-19 vaccine response in patients with non-dialysis-dependent CKD (NDD-CKD).^12-15^ In addition, most of these studies looked at the response to 2 doses and were conducted prior to the emergence of some of the more novel variants of concerns, including Omicron (B.1.1.529). One study reported anti-spike levels to be slightly reduced but comparable to healthy controls after a 2-dose regimen of the mRNA-1273 vaccine.^13^ There is scarce information regarding the response to 3 doses of mRNA COVID-19 vaccines in NDD-CKD patients. Understanding the response to COVID-19 vaccines in the NDD-CKD population is paramount as these patients have higher hospitalization and fatality rates from COVID-19.^16,17^ In this prospective cohort study, our objective was to describe the serologic response over time, following a third mRNA COVID-19 vaccine dose in NDD-CKD patients, and to examine its association with demographic and clinical factors. Knowledge from this study will help to inform future COVID-19 vaccination strategies in this population, including frequency of dosing.

## Materials and Methods

### Study Design and Population

Participants were recruited from outpatient kidney clinics within Ontario and British Columbia, Canada for this prospective observational cohort study. The NDD-CKD (G1-G5) patients aged ≥18 years, including those on immunosuppressive treatment, who received 3 doses of BNT162b2 (Pfizer-BioNTech) and/or mRNA-1273 (Moderna) met the inclusion criteria. Patients who initiated maintenance dialysis or received a kidney transplant during the study period were excluded. Between August 27, 2021, and November 30, 2022, blood samples were collected for serological testing every 3 months, as either dried blood spot or serum samples based on feasibility between 14 days and 9 months after the third dose. Demographics, comorbidities, medications, laboratory values, and hospital admissions were obtained from the Ontario Renal Reporting System (ORRS) and Patient Records and Outcome Management Information System (PROMIS) in Ontario and British Columbia, respectively.

### Serology Measurements

Detection of the wild-type strain of SARS-CoV-2-specific immunoglobulin G (IgG) binding antibodies directed against the full-length spike protein (anti-spike), the anti-receptor binding domain (anti-RBD) of the spike protein, and the anti-nucleocapsid protein (anti-NP) was performed on a custom-automated enzyme-linked immunosorbent assay (ELISA) platform previously described.^18-20^ The NP is not an antigen target of mRNA COVID-19 vaccines and thus anti-NP levels were used to infer prior COVID-19.

The levels of antibodies to each of the antigens were normalized to reference standards included on each plate (in collaboration with the National Research Council of Canada) and expressed in binding antibody units per milliliter (BAU/mL), the World Health Organization International Standard unit for anti-SARS-CoV-2 immunoglobulin. Thresholds for positivity (seropositivity) were defined as 3 SDs from the mean of negative controls. Seropositivity thresholds in BAU/mL are 11.28, 30.97, and 34.46 for anti-spike, anti-RBD, and anti-NP antibodies, respectively.

The level of IgG antibodies in convalescent serum from patients who have recovered from COVID-19 have been considered to be a useful comparator for vaccine immunogenicity as vaccinated individuals should be expected to, at minimum, reach the levels seen in those with prior COVID-19.^21,22^ Thus, we utilized the median antibody levels from convalescent serum as a marker of a robust antibody response; we used the antibody levels determined from convalescent sera obtained 21 to 115 days post-symptom onset from a cohort of 211 COVID-19-infected patients with mild to severe presentation and a median age of 59 years.^20^ Anti-NP and anti-RBD (n=211) were analyzed at 1:160 and 1:640, n=80 (subset of the 211) for anti-spike were analyzed at 1:160 and 1:2560.^20^ The median convalescent serum levels in BAU/mL are 561, 459, and 123 for anti-spike, anti-RBD, and anti-NP antibodies, respectively.

### Statistical Methods

The participants’ demographic and clinical characteristics were expressed as count (%) for categorical variables and median (interquartile range [IQR]) for continuous variables. Samples were grouped into monthly intervals and stratified by anti-NP seroconversion status. The proportion of participants who seroconverted or achieved convalescent levels was summarized by monthly interval and over the follow-up duration. Univariable and multivariable logistic regression analyses were used to evaluate the association between mounting a robust antibody response and key covariables of interest. A quadratic regression model was used to examine the relationship between IgG antibody levels and time. Within-participant correlation was accounted for in the covariance structure of the random errors. Interaction terms between the covariables of interest and time were built into the models to examine the variability of this relationship by different levels of the covariables.

We selected the following covariables based upon factors that might plausibly affect the immune response to vaccination in this population: age, sex, eGFR, urine albumin-creatinine ratio (ACR), cause of CKD, and immunosuppressive treatment. Each covariable was assessed one at a time while adjusting for all the other covariables. Recognizing there could be interactions between the covariables, we also examined the interactions of eGFR × ACR, and Age × Sex, on antibody response over time. Age was stratified into tertiles (<58, 58-73, and ≥73 years), eGFR was dichotomous as less than or ≥30 mL/min per 1.73 m^2^, and the KDGIO categories for ACR were used (A1: <3 mg/mmol, A2: 3-30 mg/mmol, and A3: >30 mg/mmol). The cause of CKD was categorized as glomerulonephritis (GN) without immunosuppressive treatment, diabetic nephropathy (DN), hypertensive nephropathy, and other. Immunosuppressive treatment included azathioprine, cyclosporine, cyclophosphamide, mycophenolate mofetil (MMF), prednisone, tacrolimus, and rituximab. For missing data, we conducted complete case analyses for the multivariable modeling; however, missing data were present in less than 5% of participants. We performed all analyses using SAS Version 9.4 (SAS Institute, Cary, North Carolina) and R Studio 2022.12.0+353 (Posit Software, PBC, Boston, Massachusetts).^23^

## Results

### Baseline Characteristics

Of the 285 NDD-CKD patients, 160 (56%) provided 1 blood sample, 79 (28%) provided 2 blood samples, and 46 (16%) provided 3 or more blood samples during the 9-month follow-up period after a third mRNA COVID-19 vaccine dose. The median age of the cohort was 67 years, 58% were men, 43% had an ACR >30 mg/mmol, 48% received BNT162b2 as their third dose, and 22% were on immunosuppressive treatment (Table 1). Participants with advanced CKD, eGFR <30 mL/min/1.73 m^2^ (G4-G5), were slightly older and were less likely to be on immunosuppressive medication. There were missing data for self-reported race/ethnicity (58/285; 20.4%), cause of CKD (2/285; 0.7%), eGFR (5/285; 1.8%), and ACR (18/285; 6.3%).

**Table 1.** Demographic and Clinical Characteristics at Baseline of Non-Dialysis-Dependent CKD Patients.

	All (n=285)	eGFR ≥ 30 mL/min/1.73 m^2^ (n=144)	eGFR < 30 mL/min/1.73 m^2^ (n=136)
Age in years, median (IQR)	67.0 (52.0-77.0)	63.5 (47.0-74.0)	71.0 [56.5, 78.0]
Male sex	166 (58.2%)	82 (56.9%)	81 (59.6%)
Race/ethnicity			
White	101 (35.4%)	45 (31.3%)	56 (41.2%)
Oriental or South Asian	26 (9.1%)	8 (5.6%)	18 (13.2%)
Other	100 (35.1%)	62 (43.1%)	36 (26.5%)
Cause of CKD			
Glomerulonephritis	100 (35.1%)	62 (43.1%)	38 (27.9%)
Diabetic nephropathy	24 (8.4%)	6 (4.2%)	18 (13.2%)
Hypertensive nephropathy	44 (15.4%)	18 (12.5%)	26 (19.1%)
Other	115 (40.3%)	58 (40.2%)	54 (39.7%)
eGFR (mL/min/1.73 m^2^)			
CKD G1	12 (4.2%)	12 (8.3%)	—
CKD G2	15 (5.3%)	15 (10.4%)	—
CKD G3a	27 (9.5%)	27 (18.8%)	—
CKD G3b	90 (31.6%)	90 (62.5%)	—
CKD G4	111 (38.9%)	—	111 (81.6%)
CKD G5	25 (8.8%)	—	25 (18.4%)
Missing	5 (1.8%)	—	—
ACR (mg/mmol)			
ACR A1 (<3)	60 (21.1%)	40 (27.8%)	20 (14.7%)
ACR A2 (3-30)	86 (30.2%)	47 (32.6%)	39 (28.7%)
ACR A3 (>30)	121 (42.5%)	48 (33.3%)	73 (53.7%)
Missing	18 (6.3%)	9 (6.3%)	4 (2.9%)
Immunosuppressive treatment	62 (21.8%)	44 (30.6%)	18 (13.2%)
Mycophenolic acid	18 (29%)	12 (27%)	6 (29%)
Prednisone	36 (58%)	26 (59%)	10 (55%)
Rituximab	3 (5%)	3 (7%)-	0 (0%)
Tacrolimus	16 (26%)	14 (32%)	2 (11%)
Azathioprine	6 (10%)	5 (11%)	1 (5%)
Cyclosporine	11 (18%)	8 (18%)	3 (17%)
Cyclophosphamide	1 (2%)	1 (2%)	1 (5%)
Number of immunosuppressive medications			
1	34 (55%)	21 (48%)	13 (72%)
2	26 (42%)	21 (48%)	5 (28%)
3	2 (3%)	2 (4%)	0 (0%)
Vaccine brand (first/second dose)			
Pfizer (BNT 162b2) only	197 (69.1%)	102 (70.8%)	91 (66.9%)
Moderna (mRNA-1273) only	30 (10.5%)	16 (11.1%)	14 (10.3%)
Mixed	42 (14.7%)	18 (12.5%)	24 (17.6%)
Vaccine brand (third dose)			
Pfizer (BNT 162b2)	137 (48.1%)	73 (50.7%)	60 (44.1%)
Moderna (mRNA-1273)	147 (51.6%)	70 (48.6%)	76 (55.9%)

*Note*. ACR = albumin-creatinine ratio; CKD = chronic kidney disease; eGFR = estimated glomerular filtration rate; IQR = interquartile range.

### SARS-CoV-2 IgG Binding Antibody Response

Anti-spike and anti-RBD levels ranged from 0.4 to 1501 and from 0.9 to 4454 BAU/mL, respectively, following a third mRNA COVID-19 vaccine dose over a 9-month follow-up period. In our cohort, anti-spike and anti-RBD levels peaked at 2 months after the third dose, with geometric mean levels at 1131 and 1672 BAU/mL, respectively, and a gradual decrement in antibody levels over time was observed (Table 2). The seropositivity rates for anti-spike and anti-RBD ranged from 85% to 100%, and 37 patients had at least 1 anti-NP seropositive sample, which suggests a prior infection with SARS-CoV-2. The rates of seropositivity at each time point did not significantly change after excluding patients with anti-NP positive samples from our analysis (Table 2 and Supplemental Table S1). The percentage of participants with a robust anti-spike and anti-RBD response, defined as reaching the convalescent serum levels, ranged from 44% to 86% over the 9-month follow-up period.

**Table 2.** Geometric Mean Antibody Levels, Seropositivity Rates, and Proportion of Patients Attaining Median Convalescent Serum Levels After a Third mRNA COVID-19 Vaccine Dose on Samples Seronegative for Anti-NP.

Time point after dose 3	Geometric mean antibody levels in BAU/mL^a^ (SD)		Proportion of seropositive participants^b^		Proportion of participants who reached convalescent level^c^	
	Anti-spike	Anti-RBD	Anti-spike (%)	Anti-RBD (%)	Anti-spike (%)	Anti-RBD (%)
1 month (n=47)	744 (6.98)	1039 (9.05)	94	89	85	81
2 months (n=29)	1131 (2.48)	1672 (3.42)	100	97	86	90
3 months (n=48)	768 (4.35)	617 (7.65)	96	90	79	63
4 months (n=44)	675 (4.35)	489 (6.41)	98	91	66	61
5 months (n=41)	479 (6.95)	356 (6.88)	93	85	68	56
6 months (n=52)	453 (8.79)	447 (9.62)	90	87	69	54
7 months (n=34)	369 (7.02)	337 (8.31)	94	91	56	44
8 months (n=21)	559 (3.22)	637 (4.48)	100	100	57	48
9 months (n=16)	426 (6.8)	461 (8)	88	88	56	56

*Note*. BAU/mL = binding antibody units per milliliter; NP = nucleocapsid protein; RBD = receptor binding domain; SARS-CoV-2 = severe acute respiratory syndrome coronavirus 2.

a World Health Organization International Standard units for anti-SARS-CoV-2 immunoglobulin: BAU/mL.

b Seropositivity threshold levels represented a positive test and the BAU/mL units are 11.28, 30.97, and 34.46 for anti-spike, anti-RBD, and anti-nucleocapsid, respectively.

c The median levels of antigen in convalescent serum that were measured 21 to 115 days post-symptom onset in a cohort of 211 adults with mild to moderate COVID-19 presentation represented a robust antibody response: 459, 561, and 123 BAU/mL for anti-RBD, anti-spike, and anti-nucleocapsid, respectively.

### Factors Associated With a Robust SARS-CoV-2 IgG Binding Antibody Response

Immunosuppressive treatment was associated with a lower likelihood of mounting a robust anti-spike response in both univariable (odds ratio [OR] 0.43, 95% confidence interval [CI]: 0.20, 0.93) and multivariable (OR 0.36, 95% CI: 0.14, 0.92) analyses. However, the association between immunosuppression and mounting a robust anti-RBD response was nonsignificant in both univariable (OR 0.52, 95% CI: 0.25, 1.10) and multivariable (OR 0.36, 95% CI: 0.14, 0.92) models. Age, sex, eGFR, ACR, and cause of CKD, including GN patients not on immunosuppressive treatment, were not found to be significantly associated with mounting a robust anti-spike or anti-RBD response in both the univariate and multivariate logistic regression analyses (Table 3).

**Table 3.** Univariate and Multivariate Logistic Regression Analysis on Factors Influencing Achieving a Robust^a^ Anti-Spike and Anti-RBD Response After the Third COVID-19 Dose.

(A) Outcome: robust anti-spike response				
Variable	Univariable		Multivariable	
	OR [95% CI]	*P* value	OR [95% CI]	*P* value
eGFR^b^ <30 (vs eGFR ≥30)	0.77 [0.39, 1.52]	.45	0.69 [0.33, 1.45]	.33
ACR^b^		.99		.99
ACR A2 (vs ACR A1)	1.06 [0.43, 2.58]		1.05 [0.41, 2.70]	
ACR A3 (vs ACR A1)	1.02 [0.44, 2.38]		1.06 [0.39, 2.88]	
Age,^c^ years		.54		.36
Age 58-73 (vs age <58]	0.65 [0.25, 1.69]		0.57 [0.20, 1.57]	
Age ≥73 (vs age <58)	0.61 [0.25, 1.49]		0.47 [0.16, 1.35]	
Male^c^ (vs female)	0.79 [0.39, 1.59]	.51	0.77 [0.36, 1.66]	.50
Immunosuppressive treatment (vs no)	0.43 [0.20, 0.93]	.03	0.36 [0.14, 0.92]	.03
(A) Outcome: robust anti-spike response				
Variable	Univariable		Multivariable	
	OR [95% CI]	*P* value	OR [95% CI]	*P* value
Cause of CKD		.53		.71
Diabetic nephropathy (vs glomerulonephritis)	2.75 [0.54, 13.92]		2.44 [0.44, 13.61]	
Hypertensive nephropathy (vs glomerulonephritis)	1.15 [0.44, 2.99]		1.07 [0.33, 3.48]	
Others (vs glomerulonephritis)	1.56 [0.70, 3.47]		1.39 [0.51, 3.78]	
(B) Outcome: robust anti-RBD response				
Variable	Univariable		Multivariable	
	OR [95% CI]	*P* value	OR [95% CI]	*P* value
eGFR^d^ <30 (vs eGFR ≥30)	0.63 [0.33, 1.19]	.15	0.69 [0.33, 1.45]	.08
ACR^d^		.95		.96
ACR A2 (vs ACR A1)	0.96 [0.42, 2.22]		1.05 [0.41, 2.70]	
ACR A3 (vs ACR A1)	1.00 [0.45, 2.23]		1.06 [0.39, 2.88]	
Age,^e^ years		.74		.77
Age 58-73 (vs age <58)	0.75 [0.32, 1.78]		0.57 [0.20, 1.57]	
Age ≥73 (vs age <58)	0.74 [0.33, 1.65]		0.47 [0.16, 1.35]	
Male^e^ (vs female)	0.70 [0.37, 1.35]	.29	0.77 [0.36, 1.66]	.22
Immunosuppressive treatment (vs no)	0.52 [0.25, 1.10]	.09	0.36 [0.14, 0.92]	.07
Cause of CKD		.43		.46
Diabetic nephropathy (vs glomerulonephritis)	3.60 [0.72, 18.10]		2.44 [0.44, 13.61]	
Hypertensive nephropathy (vs glomerulonephritis)	1.01 [0.41, 2.50]		1.07 [0.33, 3.48]	
Others (vs glomerulonephritis)	1.28 [0.61, 2.73]		1.39 [0.51, 3.78]	

*Note*. ACR = albumin-creatinine ratio; CI = confidence interval; CKD = CKD = chronic kidney disease; eGFR = estimated glomerular filtration rate; OR = odds ratio; RBD = receptor binding domain.

a The median levels of antigen in convalescent serum that were measured 21 to 115 days post-symptom onset in a cohort of 211 adults with mild to moderate COVID-19 presentation represented a robust antibody response: 459, 561, and 123 BAU/mL for anti-RBD, anti-spike, and anti-nucleocapsid, respectively.

b No interaction between eGFR and ACR on the probability of mounting response for anti-spike (univariable *P* value = .71, multivariable *P* value = .79).

c No interaction between age and sex on the probability of mounting response for anti-spike (univariable *P* value = .62, multivariable *P* value = .72).

d No interaction between eGFR and ACR on the probability of mounting response for anti-RBD (univariable *P* value = .38, multivariable *P* value = .53).

e No interaction between age and sex on the probability of mounting response for anti-RBD (univariable *P* value = .74, multivariable *P* value = .90).

### Factors Associated With SARS-CoV-2 IgG Binding Antibodies Over Time

**Figure 1.**
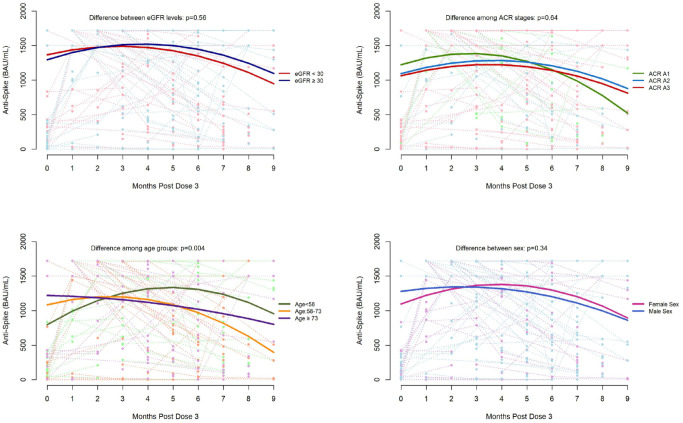
SARS-CoV-2 immunoglobulin G spike antibody response in non-dialysis-dependent (G1-G5) chronic kidney disease patients up to 9 months after a third dose of mRNA COVID-19 vaccination by eGFR, urine ACR, age, and sex. *Note*. Dots represent individual blood samples. Solid line indicates median levels. Dashed line connects samples from the same participant at different time points. ACR = albumin-creatinine ratio; eGFR = estimated glomerular filtration rate; SARS-CoV-2 = severe acute respiratory syndrome coronavirus 2.

**Figure 2.**
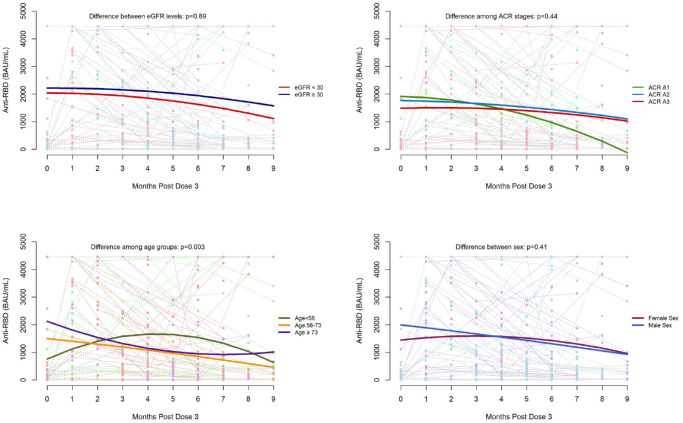
SARS-CoV-2 immunoglobulin G RBD antibody response in non-dialysis-dependent (G1-G5) chronic kidney disease patients up to 9 months after a third dose of mRNA COVID-19 vaccination by eGFR, urine ACR, age, and sex. *Note*. Dots represent individual blood samples. Solid line indicates median levels. Dashed line connects samples from the same participant at different time points. ACR = albumin-creatinine ratio; eGFR = estimated glomerular filtration rate; RBD = receptor binding domain; SARS-CoV-2 = severe acute respiratory syndrome coronavirus 2.

After the third dose, the relationship between anti-spike and time was found to vary by age groups (<58, 58-73, and ≥73 years) in both unadjusted (*P* = .005) and adjusted (*P* = .004) analyses (Figure 1). The relationship between anti-RBD and time also differed by age groups in both unadjusted (*P* = .028) and adjusted (*P* = .026) analyses (Figure 2). Age and sex interacted with the relationship between anti-spike levels and time (unadjusted *P* = .017, adjusted *P* = .007). (Supplemental Figure S1). There was no difference in the relationship between IgG antibody levels and time by sex, eGFR, ACR, cause of CKD, and immunosuppression in both adjusted and unadjusted models (Supplemental Figures S1 and S2).

## Discussion

In this study, we investigated the humoral immune response to 3 doses of mRNA COVID-19 vaccines in NDD-CKD (G1-G5) patients. We found that almost all participants were seropositive for anti-spike and anti-RBD SARS-CoV-2 IgG antibodies following the third mRNA COVID-19 vaccine dose. The high seropositive rates observed are in accordance with results from two other studies with a total of 75 NDD-CKD patients, with seroconversion rates of 96% to 97% for IgG SARS-CoV-2 antibodies, following the third COVID-19 vaccine dose.^24,25^ We observed a decline in antibody levels over time, which is consistent with the known phenomenon of waning of antibody levels post-COVID-19 vaccination in both the general population and in CKD patients.^25,26^ However, despite a gradual decrease in antibody levels over the 9-month follow-up period, reassuringly the proportion of participants with detectable COVID-19 antibodies remained relatively unchanged.

Prior studies have demonstrated that the humoral response to vaccines decreases with declining eGFR in NDD-CKD patients. For example, DaRosa et al,^9^ showed that CKD stage predicts seroconversion after hepatitis B immunization and Hashemi et al^27^ showed a significant positive correlation between hepatitis B antibody levels and eGFR. We analyzed eGFR both as a dichotomous and continuous variable and found no significant association between eGFR and SARS-CoV-2 antibody levels over time or with mounting a robust response. This finding remained unchanged when examining the impact of urine ACR and the potential interaction between eGFR and ACR. The association between antibody response and eGFR in NDD-CKD patients after hepatitis B immunization, but not after COVID-19 immunization, along with high seropositivity rates observed in the latter, could be potentially attributed to differences in vaccine technology. Compared with other vaccine types, mRNA vaccines induce both antibody and CD8+ T cell responses, directly interact with pattern recognition receptors, and do not require adjuvants.^28^ The magnitude of the immune response elicited by mRNA vaccines has led to the production and testing of mRNA vaccines against a variety of infectious diseases.^29,30^ This observation is of interest and warrants future studies of mRNA vaccines against hepatitis B, influenza, and other infectious diseases in the CKD population to determine whether these will confer more robust immune response in comparison with older vaccines that require frequent “boosting” to waning immunity.

There are many factors that can influence the antibody response to COVID-19 vaccination, such as age, sex, comorbidities, or medications. Several studies have reported that older age, male sex, increasing number of comorbidities, and medications, such as immunosuppressive drugs, can blunt the response to COVID-19 vaccines in the general population.^31-34^ We found that the relationship between SARS-CoV-2 IgG antibody levels and time varied with age in NDD-CKD patients, which suggests that the decrease in antibody levels over time occurs faster in older patients. These observations support policies that older individuals may need more frequent COVID-19 booster doses.^35,36^ We also observed an interaction between age and sex on antibody response over time; older men had a steeper antibody decline over time compared with younger women. We found that NDD-CKD patients on immunosuppressive treatment were less likely to mount a robust anti-spike IgG antibody response. The negative effect of immunosuppressive drugs on the immune response to mRNA COVID-19 vaccination in NDD-CKD patients have also been reported by a few small studies with G4-G5 patients.^14,24^ Interestingly, we also observed that NDD-CKD patients with GN, but not on immunosuppressive medication, did not have a significantly different antibody response to the COVID-19 vaccines compared with patients with other causes of kidney disease. This suggests that the underlying causes of CKD, even those that are considered to be autoimmune in nature, did not meaningfully impact antibody levels.

This study has several strengths. First, serological testing was performed on samples collected at multiple time points after the third dose. As there is limited information on the humoral response to COVID-19 vaccines in NDD-CKD patients, especially after the third dose, our study provides some insights into the magnitude and duration of antibody responses months after vaccination in this population. Second, antibody levels were reported in WHO units, BAU/mL, which allow for standardization and comparison of results in studies that used different assays. Third, we used a set of standardized, high-throughput serological assays that can detect 3 different antibodies against SARS-CoV-2 and have been used in more than 40 clinical studies, making it possible to harmonize and compare results from other cohorts. Fourth, unlike many other studies, we accounted for prior asymptomatic COVID-19 infection in our analysis by measuring anti-NP levels. And finally, we had a diverse CKD population, with patients with a variety of disease etiologies, age, and racial backgrounds.

This study also has limitations. First, given the rapid pace of vaccination, we were unable to obtain samples prior to the first 2 doses to establish baseline SARS-CoV-2 IgG antibody levels. This hindered our ability to precisely determine the influence of the third dose alone on the overall serologic response. For instance, we could not measure the extent to which the third dose increased antibody levels compared with 1 or 2 doses, or its potential to seroconvert individuals who were seronegative after 2 doses. Second, our study also encountered challenges in obtaining blood samples from all participants at every scheduled time point. Some participants changed their vaccine dose status by receiving a fourth dose approximately 3 to 6 months after the third, and individuals who opted to mail dried blood spot samples sometimes experienced delays or forgot to do so. Third, as vaccinated individuals tend to generate fewer anti-NP antibodies after infection,^37,38^ the reported proportion of our cohort with prior COVID-19 may be an underestimate as a lower threshold for positivity is required. Fourth, neutralizing antibodies and cellular immune responses were not evaluated in this study although our prior work has shown a strong correlation between neutralizing antibody responses and binding IgG antibody response.^39^ In addition, there remains no established antibody threshold for protection. While binding antibodies measured in this study correlate well with neutralizing antibodies against Omicron variants of concern,^40^ previous work has shown that, in patients with end-stage kidney disease, neutralizing antibodies were 48.2-fold lower against the XBB.1.5 Omicron subvariant in comparison with the original SARS-CoV-2 Wuhan strain.^41^ Given the ongoing evolution of SARS-CoV-2 and Omicron subvariants that have the potential for immune escape, in the future it will be prudent to conduct serosurveillance studies against emerging SARS-CoV-2 subvariants to understand the protection conferred by additional vaccines doses. Finally, we did not evaluate the relationship between SARS-CoV-2 IgG antibody levels and clinical outcomes, such as rate of infection and hospitalization, as they were minimal during the study period.

In conclusion, patients with NDD-CKD exhibit a strong humoral immune response following a third mRNA COVID-19 vaccine dose and their SARS-CoV-2 IgG antibody responses are not associated with eGFR or albuminuria.

## Supplemental Material

sj-docx-3-cjk-10.1177_20543581231224127 – Supplemental material for Humoral Response Following 3 Doses of mRNA COVID-19 Vaccines in Patients With Non-Dialysis-Dependent CKD: An Observational Study Click here for additional data file. Supplemental material, sj-docx-3-cjk-10.1177_20543581231224127 for Humoral Response Following 3 Doses of mRNA COVID-19 Vaccines in Patients With Non-Dialysis-Dependent CKD: An Observational Study by Omosomi Enilama, Kevin Yau, Lee Er, Mohammad Atiquzzaman, Matthew J. Oliver, Marc G. Romney, Jerome A. Leis, Kento T. Abe, Freda Qi, Karen Colwill, Anne-Claude Gingras, Michelle A. Hladunewich and Adeera Levin in Canadian Journal of Kidney Health and Disease

sj-jpeg-1-cjk-10.1177_20543581231224127 – Supplemental material for Humoral Response Following 3 Doses of mRNA COVID-19 Vaccines in Patients With Non-Dialysis-Dependent CKD: An Observational Study Click here for additional data file. Supplemental material, sj-jpeg-1-cjk-10.1177_20543581231224127 for Humoral Response Following 3 Doses of mRNA COVID-19 Vaccines in Patients With Non-Dialysis-Dependent CKD: An Observational Study by Omosomi Enilama, Kevin Yau, Lee Er, Mohammad Atiquzzaman, Matthew J. Oliver, Marc G. Romney, Jerome A. Leis, Kento T. Abe, Freda Qi, Karen Colwill, Anne-Claude Gingras, Michelle A. Hladunewich and Adeera Levin in Canadian Journal of Kidney Health and Disease

sj-jpeg-2-cjk-10.1177_20543581231224127 – Supplemental material for Humoral Response Following 3 Doses of mRNA COVID-19 Vaccines in Patients With Non-Dialysis-Dependent CKD: An Observational Study Click here for additional data file. Supplemental material, sj-jpeg-2-cjk-10.1177_20543581231224127 for Humoral Response Following 3 Doses of mRNA COVID-19 Vaccines in Patients With Non-Dialysis-Dependent CKD: An Observational Study by Omosomi Enilama, Kevin Yau, Lee Er, Mohammad Atiquzzaman, Matthew J. Oliver, Marc G. Romney, Jerome A. Leis, Kento T. Abe, Freda Qi, Karen Colwill, Anne-Claude Gingras, Michelle A. Hladunewich and Adeera Levin in Canadian Journal of Kidney Health and Disease
