# Supplementary material for: Humoral Response Following 3 Doses of mRNA COVID-19 Vaccines in Patients With Non-Dialysis-Dependent CKD: An Observational Study
Source: Can J Kidney Health Dis. 2024 Jan 29;11:20543581231224127. doi: 10.1177/20543581231224127 (PMC10826386; doi:10.1177/20543581231224127)
Supplement: sj-docx-3-cjk-10.1177_20543581231224127 – Supplemental material for Humoral Response Following 3 Doses of mRNA COVID-19 Vaccines in Patients With Non-Dialysis-Dependent CKD: An Observational Study [file sj-docx-3-cjk-10.1177_20543581231224127.docx]

**Supplementary Materials**

**Supplementary Figure 1:** SARS-CoV-2 immunoglobulin G Spike antibody response in non-dialysis-dependent (G1-G5) chronic kidney disease (CKD) patients up to 9 months after a third dose of mRNA COVID-19 vaccination by immunosuppressive treatment status, cause of CKD, the combination of eGFR and urine albumin-creatinine ratio (ACR), and the combination of age and sex. Dots represent individual blood samples. Solid line indicates median levels. Dashed line connects samples from the same participant at different timepoints.

**Supplementary Figure 2:** SARS-CoV-2 immunoglobulin G RBD antibody response in non-dialysis-dependent (G1-G5) chronic kidney disease (CKD) patients up to 9 months after a third dose of mRNA COVID-19 vaccination by immunosuppression treatment status, cause of CKD, the combination of eGFR and urine albumin-creatinine ratio (ACR), and the combination of age and sex. Dots represent individual blood samples. Solid line indicates median levels. Dashed line connects samples from the same participant at different timepoints.

**Supplementary Table 1:** Geometric mean antibody titers, seropositivity rates, and proportion of patients attaining median convalescent serum levels after a third mRNA COVID-19 vaccine dose on all samples.


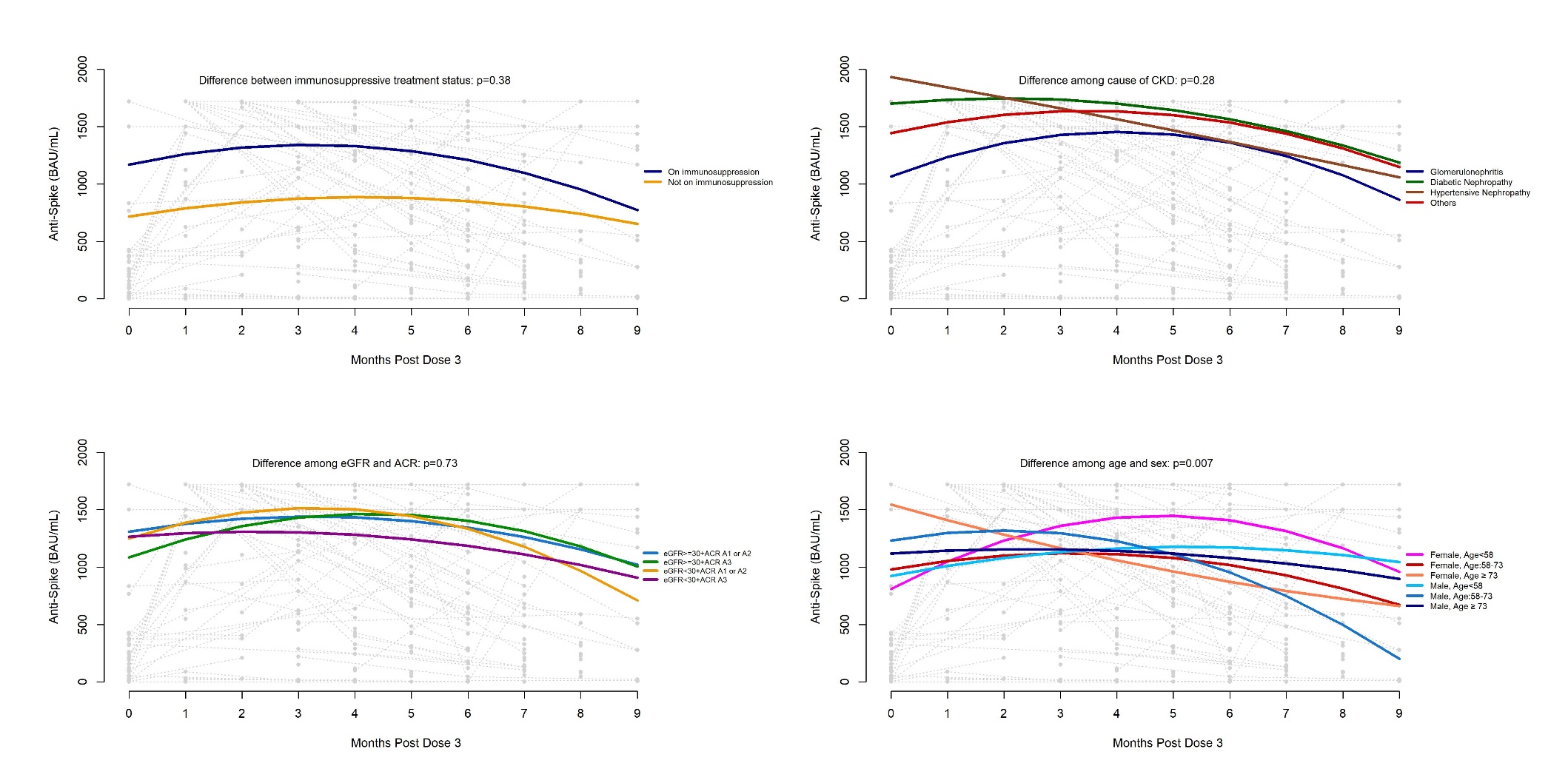


**Supplementary Figure 1: SARS-CoV-2 immunoglobulin G Spike antibody response in non-dialysis-dependent (G1-G5) chronic kidney disease (CKD) patients up to 9 months after a third dose of mRNA COVID-19 vaccination by immunosuppressive treatment status, cause of CKD, the combination of eGFR and urine albumin-creatinine ratio (ACR), and the combination of age and sex**. Dots represent individual blood samples. Solid line indicates median levels. Dashed line connects samples from the same participant at different timepoints.


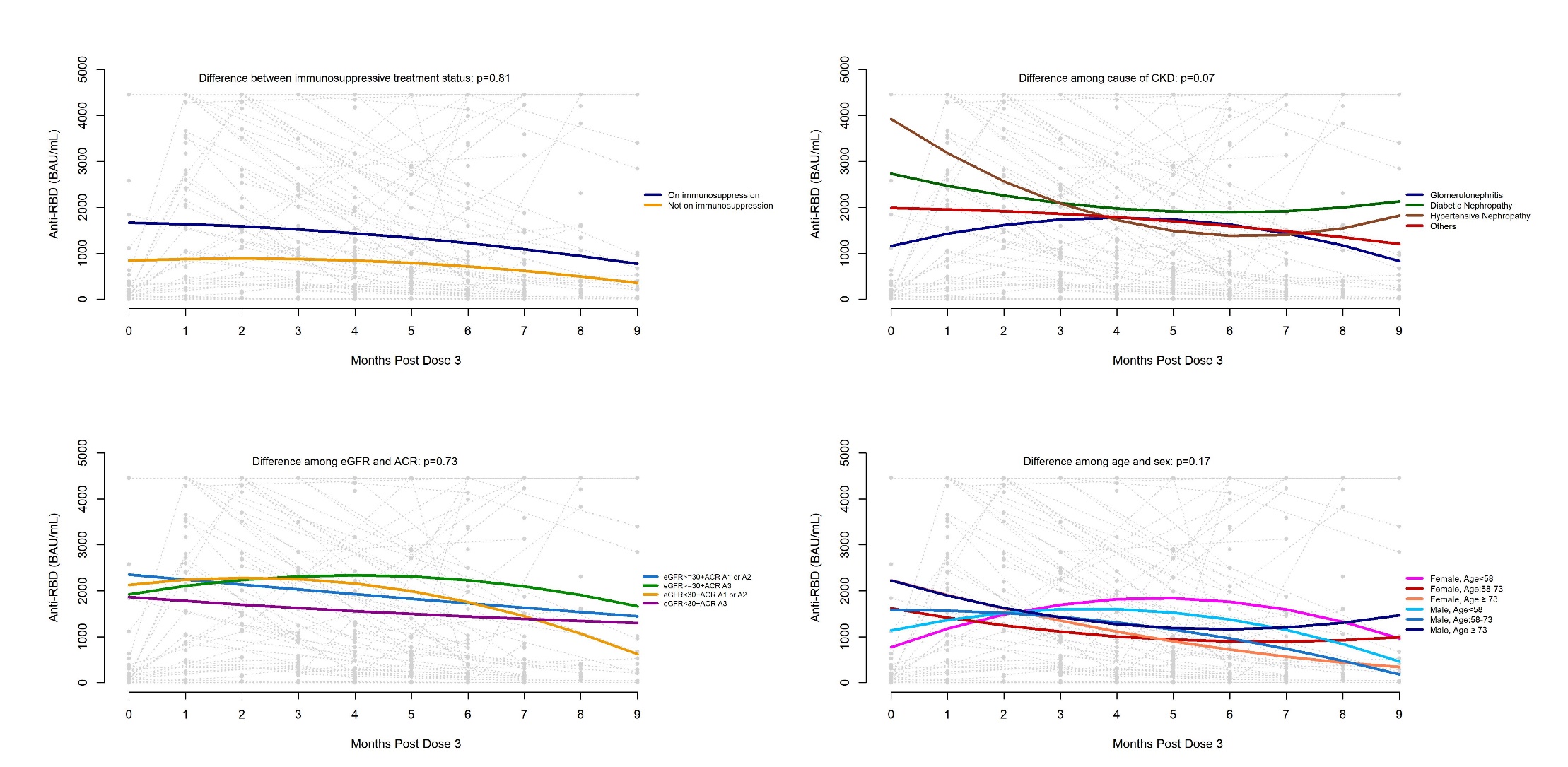


**Supplementary Figure 2: SARS-CoV-2 immunoglobulin G RBD antibody response in non-dialysis-dependent (G1-G5) chronic kidney disease (CKD) patients up to 9 months after a third dose of mRNA COVID-19 vaccination by immunosuppression treatment status, cause of CKD, the combination of eGFR and urine albumin-creatinine ratio (ACR), and the combination of age and sex.** Dots represent individual blood samples. Solid line indicates median levels. Dashed line connects samples from the same participant at different timepoints.

| **Timepoint**  **After Dose 3** | **Geometric Mean Antibody Titers in BAU/mL^[[1]](#footnote-1)^ (SD)** | | **No. (%) of seropositive participants ^[[2]](#footnote-2)^** | | **No. (%) of participants who reached Convalescent level^[[3]](#footnote-3)^** | |
| --- | --- | --- | --- | --- | --- | --- |
|  | Anti-spike | Anti-RDB | Anti-spike | Anti-RDB | Anti-spike | Anti-RDB |
| **1 month**  **(n= 47)** | 757 (6.87) | 1071 (8.92) | 94% | 90% | 85% | 81% |
| **2 months**  **(n=29)** | 1188 (2.33) | 1761 (3.23) | 100% | 97% | 88% | 91% |
| **3 months**  **(n= 48)** | 869 (3.9) | 809 (7.05) | 97% | 92% | 81% | 68% |
| **4 months**  **(n= 44)** | 781 (3.88) | 634 (5.95) | 98% | 93% | 71% | 65% |
| **5 months**  **(n= 41)** | 542 (6.45) | 438 (6.89) | 93% | 87% | 72% | 61% |
| **6 months**  **(n= 52)** | 576 (7.4) | 622 (8.74) | 92% | 89% | 75% | 62% |
| **7 months**  **(n=34)** | 483 (6.29) | 494 (8.1) | 95% | 93% | 64% | 52% |
| **8 months**  **(n=21)** | 661 (3.12) | 815 (4.44) | 100% | 100% | 64% | 56% |
| **9 months**  **(n=16)** | 757 (4.73) | 881 (6.87) | 93% | 93% | 76% | 72% |
| **Overall**  **(n=285)** | 615 (5.32) | 614 (7.67) | 96% | 91% | 74% | 69% |

**Supplementary Table 1.** Geometric mean antibody titers, seropositivity rates, and proportion of patients attaining median convalescent serum levels after a third mRNA COVID-19 vaccine dose on all samples.

1. Abbervations: NP – nucleocapsid protein, RBD - receptor binding domain

   World Health Organization International Standard units for anti-SARS-CoV-2 immunoglobulin: binding antibody units per milliliter. [↑](#footnote-ref-1)
2. Seropositivity threshold levels represented a positive test and in BAU/ml units are 11.28, 30.97, and 34.46 for anti-spike, anti-RBD, and anti-nucleocapsid, respectively. [↑](#footnote-ref-2)
3. The median levels of antigen in convalescent serum were measured 21-115 days post symptom onset in a cohort of 211 adults with mild to moderate COVID-19 presentation represented a robust antibody response: 459, 561 and 123 BAU/ml for anti-RBD, anti-spike and anti-nucleocapsid, respectively. [↑](#footnote-ref-3)
