# Supplementary figures and images for: Humoral Response Following 3 Doses of mRNA COVID-19 Vaccines in Patients With Non-Dialysis-Dependent CKD: An Observational Study
Source: Can J Kidney Health Dis. 2024 Jan 29;11:20543581231224127. doi: 10.1177/20543581231224127 (PMC10826386; doi:10.1177/20543581231224127)

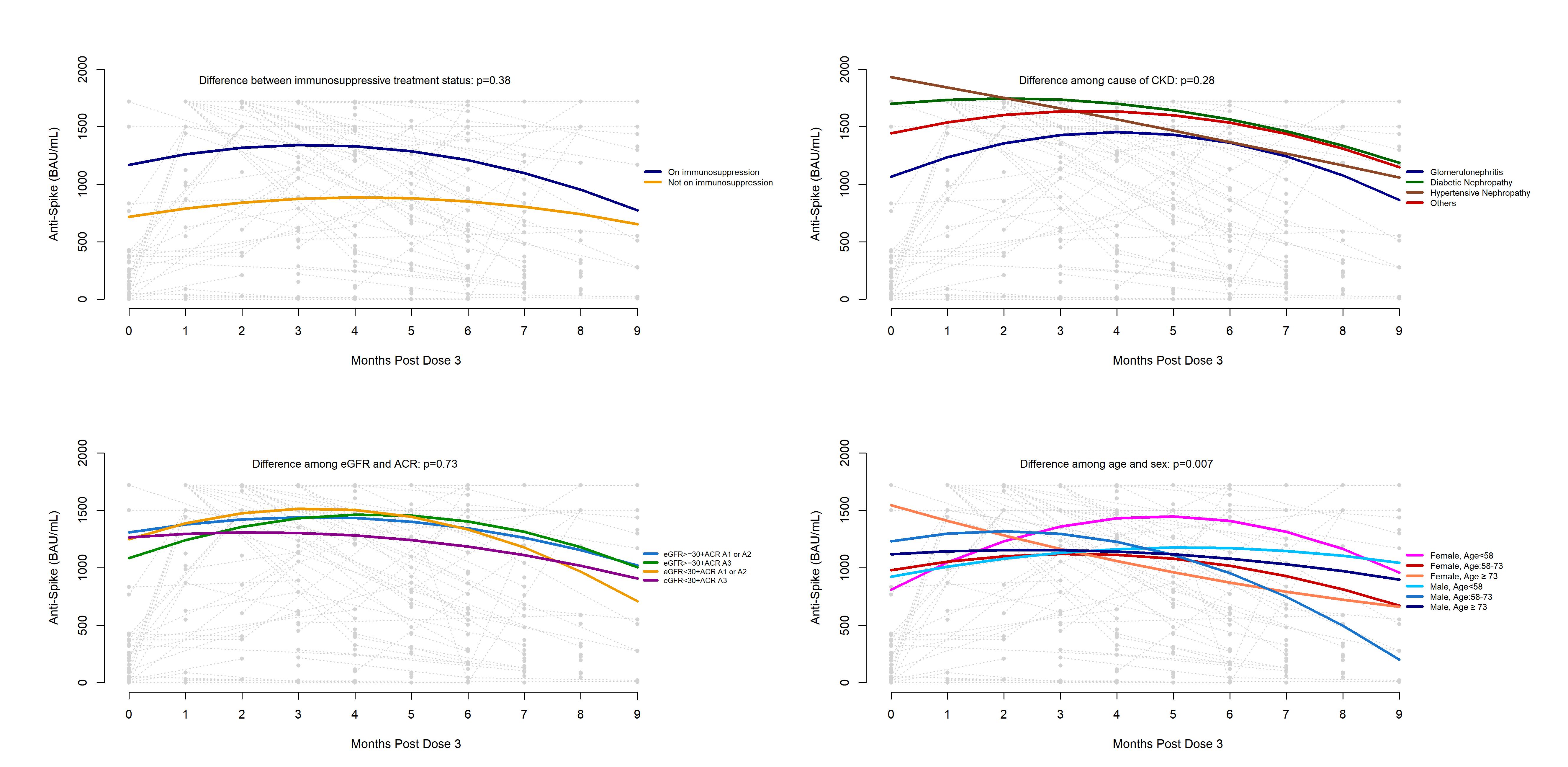

Supplement: sj-jpeg-1-cjk-10.1177_20543581231224127 – Supplemental material for Humoral Response Following 3 Doses of mRNA COVID-19 Vaccines in Patients With Non-Dialysis-Dependent CKD: An Observational Study [file sj-jpeg-1-cjk-10.1177_20543581231224127.jpeg]

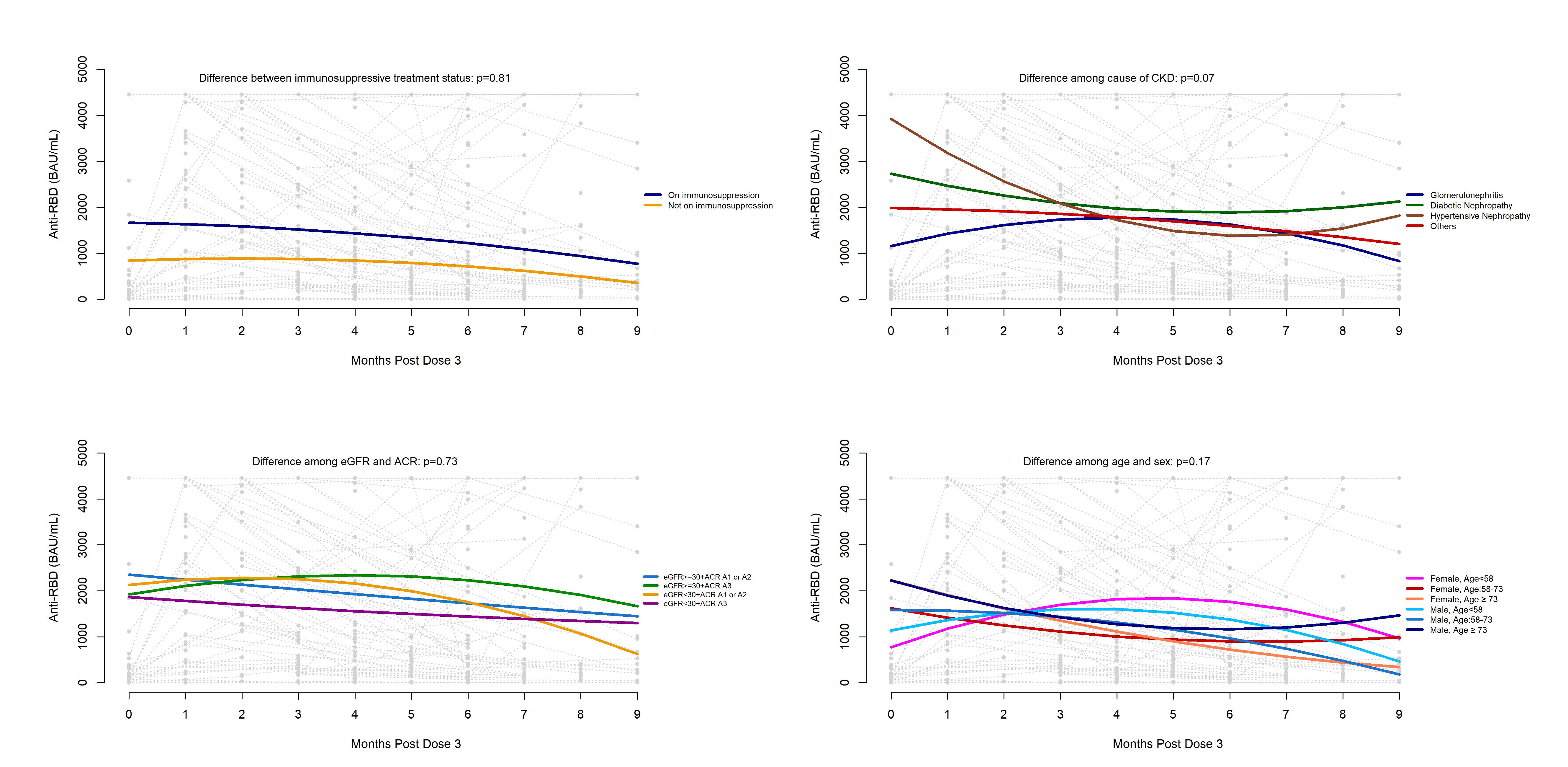

Supplement: sj-jpeg-2-cjk-10.1177_20543581231224127 – Supplemental material for Humoral Response Following 3 Doses of mRNA COVID-19 Vaccines in Patients With Non-Dialysis-Dependent CKD: An Observational Study [file sj-jpeg-2-cjk-10.1177_20543581231224127.jpeg]
